# Wyburn‐Mason Syndrome: A Case Report

**DOI:** 10.1002/kjm2.70216

**Published:** 2026-04-10

**Authors:** Yu‐Wen Wang, Chen‐Lu Pei, Jian‐Yang Gong

**Affiliations:** ^1^ The Department of Ophthalmology The First Affiliated Hospital of Anhui Medical University Hefei China; ^2^ The Department of Ophthalmology The First Affiliated Hospital of Anhui Medical University, North District Hefei China

1

Wyburn‐Mason syndrome, also known as Bonnet–Dechaume–Blanc syndrome, is a rare, congenital, and sporadic neurocutaneous disorder classified as a phakomatosis, with no clear racial or sex predilection [1]. It is characterized by multiple AVMs that most commonly involve the CNS, the retina, and, less frequently, the facial region and typically presents unilaterally. The syndrome was first systematically described by Wyburn‐Mason, who reported nine cases and defined it as a distinct clinical entity [2].

A 26‐year‐old man with progressive visual impairment in his right eye presented to the department of ophthalmology. The best‐corrected visual acuity (BCVA) was 20/200 in the right eye (OD) and 20/20 in the left eye (OS).

Scanning laser ophthalmoscopy (SLO) provided high‐resolution visualization of the retinal vasculature. It demonstrated abnormal vascular morphology characterized by irregular vessel calibers and direct arteriovenous shunts. Compared with conventional fundus photography, SLO provided enhanced contrast and improved delineation of vascular relationships, facilitating assessment of the lesion extent, particularly in the peripheral retina (Figure 1A,B).

**FIGURE 1:**
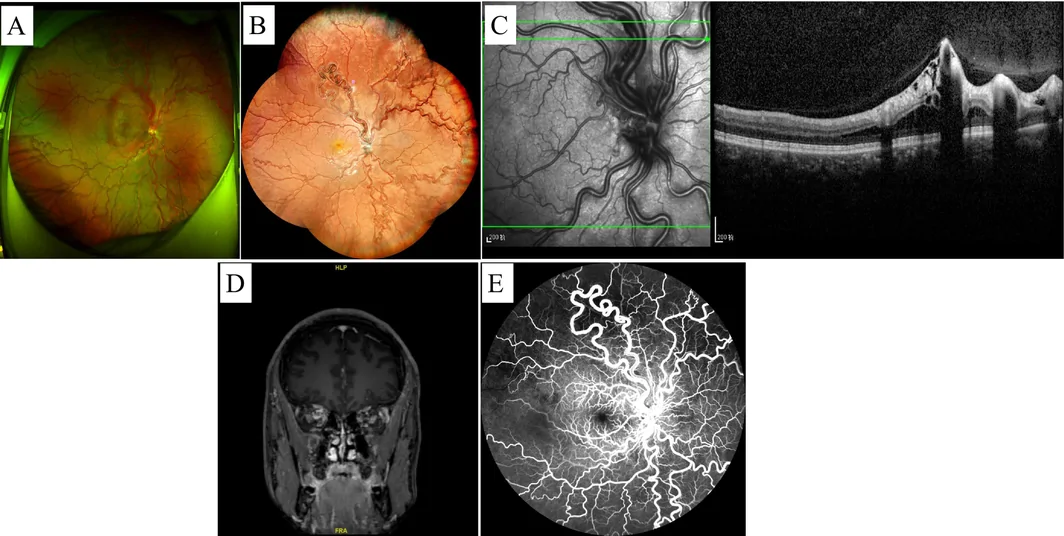
(A, B) SLO result. This ultra‐widefield fundus photograph shows markedly dilated and tortuous retinal vessels in the right eye, most prominent around the optic disc and extending toward the periphery. (C) OCT demonstrates a striking pattern of multiple circular or oval hyporeflective spaces within the retinal layers, representing cross‐sections of abnormally dilated and tortuous vessels. The surrounding retinal architecture appears displaced but relatively intact, with no evidence of cystoid macular edema or subretinal fluid. (D) Cranial MRI showed that there were multiple tortuous dilations of blood vessels along the optic nerve, extending to the skull, and the right optic nerve was not clearly demarcated, and there were no obvious abnormal enhancement foci in the left orbit, brain parenchyma and meninges. (E) The FFA image shows markedly dilated and tortuous retinal vessels, with arteries and veins filling simultaneously during the arterial phase. Some vessels appear enlarged and serpentine, extending toward the periphery and forming abnormal vascular loops. The macular region exhibits mild hypofluorescence, with no significant fluorescein leakage observed in the late phase.

Optical coherence tomography (OCT) revealed cross‐sectional structural alterations associated with the retinal AVM. Enlarged circular vascular profiles were observed within the inner retinal layers, corresponding to dilated arteriovenous channels. No cystoid macular edema, intraretinal hemorrhage, or subretinal fluid was detected. Outer retinal layers and photoreceptor integrity were preserved, indicating the absence of significant secondary structural damage (Figure 1C).

The patient had no cutaneous lesions or history of neurological disease. Cranial magnetic resonance imaging (MRI) demonstrated multiple tortuous and dilated vessels along the right optic nerve extending toward the skull, with poor delineation of the nerve. No abnormal enhancement was observed in the contralateral orbit, brain parenchyma, or meninges. The ventricular system, sulci, fissures, and midline structures appeared normal (Figure 1D).

Fluorescein fundus angiography (FFA) provided a dynamic hemodynamic assessment. Early simultaneous arterial and venous filling indicated high‐flow arteriovenous shunting, while the absence of late leakage excluded active neovascularization or significant vascular permeability changes. These findings were consistent with a Group 2 retinal AVM and supported conservative management (Figure 1E).

Wyburn–Mason syndrome is currently regarded as part of the spectrum of cerebrofacial arteriovenous metameric syndromes (CAMS), in which vascular malformations arise from segmental developmental disturbances of the embryonic vascular system. In CAMS type 2 (the optic pathway group), vascular anomalies may involve the retina, optic nerve, and surrounding structures. In the present case, the retinal AVM was accompanied by vascular abnormalities along the right optic nerve observed on MRI, suggesting involvement of the optic pathway vascular segment. Therefore, although extensive intracranial AVMs were not detected, the findings are consistent with an ocular‐dominant presentation within the Wyburn–Mason/CAMS spectrum rather than a purely isolated retinal AVM. However, we acknowledge that this presentation may also be interpreted as a retinal arteriovenous malformation with optic pathway involvement, reflecting the overlap between these entities within the same developmental spectrum.

Wyburn–Mason syndrome is a rare congenital cerebrofacial arteriovenous malformation disorder characterized by high‐flow vascular malformations involving the retina, brain, and facial structures. In the present case, comprehensive multimodal imaging—including SLO, OCT, FFA, and MRI—enabled detailed structural and hemodynamic evaluation of an isolated retinal arteriovenous malformation. Imaging revealed markedly dilated and tortuous vessels with direct arteriovenous shunting, circular vascular cross‐sections within the inner retina on OCT, and early simultaneous arterial and venous filling on FFA without late leakage, findings consistent with a Group 2 lesion [3]. MRI did not demonstrate extensive intracranial AVMs; however, vascular abnormalities along the optic nerve suggest involvement of the optic pathway segment, supporting classification within the Wyburn–Mason/CAMS spectrum.

This case highlights the critical role of multimodal imaging not only in establishing diagnostic accuracy but also in differentiating retinal AVMs from other vascular anomalies and documenting baseline morphology for long‐term monitoring. Conservative management was adopted due to the absence of exudation, hemorrhage, or macular complications. However, given the potential for delayed neurological involvement, regular ophthalmic follow‐up combined with periodic neuroimaging remains essential.

Wyburn–Mason syndrome is a rare vascular disorder with highly variable clinical manifestations depending on the location and extent of the vascular malformations. Because ocular findings may represent the earliest or sole manifestation, multimodal ophthalmic imaging combined with systemic neuroimaging plays a crucial role in early diagnosis and evaluation of associated intracranial lesions [4]. Regular follow‐up and multidisciplinary collaboration among ophthalmologists, neurologists, neurosurgeons, and radiologists are essential for monitoring disease progression and preventing severe complications.

## Ethics Statement

This case report was conducted in accordance with the ethical principles of the Declaration of Helsinki. Ethical approval for publication of a single anonymized case report was waived by the institutional review board of the Hospital, as individual case reports do not require full committee review according to local institutional policies.

## Conflicts of Interest

The authors declare no conflicts of interest.

## Data Availability

The data that support the findings of this study are available on request from the corresponding author. The data are not publicly available due to privacy or ethical restrictions.

## References

[1] R. Rizzo , L. Pavone , G. Pero , I. Chiaromonte , and P. Curatolo , “A Neurocutaneous Disorder With a Severe Course: Wyburn‐Mason's Syndrome,” Journal of Child Neurology 19 (2004): 908–911. 15658797

[2] R. Wyburn‐Mason , “Arteriovenous Aneurysm of Midbrain and Retina, Facial Naevi and Mental Changes,” Brain 66 (1943): 163–203.

[3] J. J. Bhattacharya , C. B. Luo , D. C. Suh , H. Alvarez , G. Rodesch , and P. Lasjaunias , “Wyburn‐Mason or Bonnet‐Dechaume‐Blanc as Cerebrofacial Arteriovenous Metameric Syndromes (CAMS). A New Concept and a New Classification,” Interventional Neuroradiology 7 (2001): 5–17. 10.1177/159101990100700101 PMC3621461 20663326

[4] C. B. Luo , P. Lasjaunias , and J. Bhattacharya , “Craniofacial Vascular Malformations in Wyburn‐Mason Syndrome,” Journal of the Chinese Medical Association 69 (2006): 575–580. 10.1016/S1726-4901(09)70332-6 17182351

